# Analysis of Extended Information Provided by Bluetooth Traffic Monitoring Systems to Enhance Short-Term Level of Service Prediction

**DOI:** 10.3390/s22124565

**Published:** 2022-06-17

**Authors:** Rubén Fernández Pozo, Ana Belén Rodríguez González, Mark Richard Wilby, Juan José Vinagre Díaz

**Affiliations:** Department of Mathematics Applied to Information and Communication Technologies, Universidad Politécnica de Madrid, 28040 Madrid, Spain; ruben.fernandez@upm.es (R.F.P.); anabelen.rodriguez@upm.es (A.B.R.G.); mark.wilby@upm.es (M.R.W.)

**Keywords:** bluetooth traffic monitoring system, traffic prediction, level of service, temporal components of traffic information

## Abstract

Bluetooth monitoring systems (BTMS) have opened a new era in traffic sensing, providing a reliable, economical, and easy-to-deploy solution to uniquely identify vehicles. Raw data from BTMS have traditionally been used to calculate travel time and origin–destination matrices. However, we could extend this to include other information like the number of vehicles or their residence times. This information, together with their temporal components, can be applied to the complex task of forecasting traffic. Level of service (LOS) prediction has opened a novel research line that fulfills the need to anticipate future traffic states, based on a standard link-based variable, accepted for both researchers and practitioners. In this paper, we incorporate BTMS’s extended variables and temporal information to an LOS classifier based on a Random Undersampling Boost algorithm, which is proven to efficiently respond to the data unbalance intrinsic to this problem. By using this approach, we achieve an overall recall of 87.2% for up to 15-min prediction horizons, reaching 96.6% predicting congestion, and improving the results for the intermediate traffic states, especially complex given their intrinsic instability. Additionally, we provide detailed analyses on the impact of temporal information on the LOS predictor’s performance, observing improvements up to a separation of 50 min between last features and prediction horizons. Furthermore, we study the predictor importance resulting from the classifiers to highlight those features contributing the most to the final achievements.

## 1. Introduction

Traffic forecasting is yet an unresolved challenge among the scientific community [1]. Both traffic management and information systems will evidently benefit from an anticipated knowledge about future traffic states, reducing congestion and allowing drivers to make informed decisions. In order to fulfill this need, research has opened two strands of work. On one hand, traffic models construct a representation of traffic based on the theoretical definition of its behavior given a set of variables describing flows and road structure. On the other hand, data-driven approaches extract inherent relations between different types of information, measured on the road, such as intensity, occupancy, or travel time. Despite the capacity of theoretical models of building cause-effect relationships, data-driven approaches show the advantage of providing accurate predictions, even when fed with incomplete data [2]. Most current technologies in this area apply several artificial intelligence functionalities including the *k* nearest neighbors (*k*-NN) as in [3,4] or neural networks like [5,6,7,8] to particular input data sets.

Data can be collected from a wide variety of sensors: inductive loops, license plate readers, Bluetooth traffic monitoring systems (BTMS), etc. Among them, BTMS gained attention during the past decade as they provide remote sensing capabilities that avoid expensive installation and maintenance tasks. BTMS uniquely identify vehicles in a certain coverage area. By using the information registered at different locations, we can calculate the representative travel time (TT) of each monitored stretch in the road network [9]. The great majority of researchers and practitioners agree that traffic must be represented by a link-based variable, among which TT appears as the best candidate. The underlying reason is that TT refers to the stretch, thus being able to provide, on its own, information about the state of traffic on a whole link. By using TT we avoid the extrapolation required to infer these same traffic states from variables like intensity, occupancy, and velocity provided by point-like sensors (mainly inductive loops). The only downside of TT is its inherent dependency on some of the link’s features like length, speed limit, etc. We can overcome this issue evolving TT to level of service (LOS) [10], given that LOS generalizes its calculation to any type of stretch regardless of its specific configuration.

In order to predict TT or LOS, approaches that use time series analysis achieve good results in situations where traffic shows recognizable patterns in time [11]. Time is an inherent component of traffic, showing a multidimensional nature. First, we can differentiate two separate time variables, departure TT (DTT) and arrival TT (ATT), depending on whether we measure the time required to cover a specific stretch from the perspective of the vehicles that started or finished it, respectively. Second, DTT is inherently delayed with respect to ATT, as we must wait until every vehicle departing from the origin gets to the destination in order to calculate the corresponding DTT value. Third, ATT is the only variable that we can measure in real time; however, traffic management requires DTT forecasts, which complicates the task. Fourth, there exists a correlation between past, current, and future traffic [2]. Fifth, this correlation also includes a spatial component as traffic in preceding and succeeding links has an effect on the link under study [12].

These “physical” traffic connections in space and time are not the only ones we need to consider. In our previous work [13], we observed that the prediction of the LOS on a northbound stretch of the urban expressway SE-30 in Seville, Spain, benefited from TT data captured on southbound stretches with no direct physical connections to the studied link. This means that data-driven approaches can extract hidden relationships among input variables. Furthermore, in addition to TT, we can also extract other variables from BTMS, that would extend the input data set for the predictors. This may lead us to conclude that the bigger the feature space, the better. The appearance of big data solutions and platforms has given direct access to new sources of data like weather or social networks, which reinforces the idea that massive amounts of data are the ultimate solution to traffic forecasting. Nevertheless, the reality is that the performance of predictors has more to do with quality than with volume [14] as variables may carry information already existent in the remaining set, thus resulting in redundancy and forcing the predictor to eliminate the accessory relations.

Given these issues, this work presents an analysis of the impact of temporal data on the performance of LOS predictions. We designed a mathematical framework to tackle this classification problem and validate the resulting predictor with empirical TT data captured by a BTMS deployed in real operation on the SE-30 and A-49 expressways. Introducing temporal information about ATTs in our original random undersampling boost (RUSBoost) classifier, we achieved a significant improvement in the recall values, especially on the intermediate LOS, which showed lower performance. Finally, we completed this study with a deep analysis of the predictor significance of each feature, which sheds light on the temporal components of traffic. Thus, the main contributions of this work are:

1. We formalize the introduction of time information into a short-time LOS predictor.
2. We propose an improved RUSBoost LOS classifier that makes use of the temporal components of traffic information, achieving an overall 88.7% success rate, which reaches 96.6% predicting congestion up to 15 min in the future.
3. We validate the performance of this LOS classifier with actual data from a BTMS in real operation in the SE-30 road in Seville, Spain.
4. We study the contribution of secondary variables provided by BTMS, such as count and residence time, to the prediction of LOS.
5. We analyze the predictor importance of the studied temporal features and extract conclusions about traffic’s temporal components.

## 2. Related Work

The prediction of future traffic states has opened a challenging research line. The scientific community has developed a whole set of methods that have currently led to different approaches based on artificial intelligence. A rigorous survey on these methods can be found in [1]. Artificial intelligence allows the learning of patterns that are replicated in time, showing some kind of standard behavior. In order to do this, the autonomous machines must be fed with data embedding representative information about different features regarding traffic.

Evidently, the first data to be contemplated are traffic variables themselves: vehicle count, intensity, occupancy, velocity, or travel time, among others. These variables are often fused as a way of exploiting complementary information provided by traffic sensors of a different nature like automatic vehicle identification (AVI) systems and counters. As illustrative examples of the recent work in this area, the authors in [15] fuse data collected by inductive loops and probe vehicles in order to estimate traffic states, while in [16,17] records in toll areas are exploited together with microwave sensor data to predict future behaviors. The advent and rapid expansion of big-data technology have opened traffic prediction to a new set of variables like weather [18] or social data [19]. However, the potential benefits of big data applied to traffic forecasting must first guarantee the quality of the data they use [14]; otherwise, an excessive number of input features may bias the learning process [20]. Some of these harmful effects have been studied in [21], which focused on data captured by inductive loops and license plate recognition systems. In addition, the fusion of new information sources involve specific issues regarding the spatial and temporal heterogeneity of data [22].

These potentially harmful effects directed research back to the original set of traffic variables. Consequently we look for features that show a relevant impact on the future behavior of traffic [23]. Future traffic states are undoubtedly linked to current and past behaviors, both in the link under study and the remaining links in the road network. Therefore, the study of temporal and spatial correlations between the values of a single representative variable like flow or TT may result in enhanced traffic predictors. At its first level, this study is supported by the existence of causality relations [24] between a specific variable and future traffic. This principle has been recently applied to flow and TT prediction. The authors in [25] analyzed correlations between flow data time series in order to reveal hidden causal dependencies. In [26] the authors included the spatial equivalent distance in their evaluation of the time correlations observed in data provided by inductive loops. In addition, the authors in [27] represented spatial and temporal information in two dimensions, which allowed them to transform a time series problem into an image analysis. Flow information can be used to estimate future TT exploiting spatio-temporal correlations among consecutive links applying an approach based on Markov chains [28]. Nonetheless, TT prediction is best performed by using data from AVI systems or probe vehicles. The authors in [29] developed a traffic speed predictor, which capitalized on the multi-dimensional time information and the bi-directional spatial information observed in taxi GPS data.

The analysis of spatio-temporal correlations in values of traditional traffic variables has recently opened a new strand of work that explores attention models [30], which aim at providing ways of interpreting the operation of deep neural networks (DNN) while improving their performance. This approach has been followed by works like [31,32,33], which propose DNN-based traffic flow predictors. Nonetheless, the correlation between flow or TT measurements and future traffic extends beyond causal relations in space and time [34]. We came up to this same conclusion in [13] where we observed how the TT prediction of a stretch in an expressway (SE-30, Seville, Spain) was improved by using TT data collected on the opposite direction, which presents negligible or non-existent physical connection with it. Consequently, variables may provide information about future traffic states not only because of their physical impact on it, but also because they are capable of describing a specific state in the whole traffic network in a particular moment in time.

In the light of the above, this work presents the following contributions:

- We take LOS as the prediction objective, given that it is a standard variable representative of future traffic states.
- We propose an enhanced RUSBoost classifier that exploits spatio-temporal correlations found in TT data.
- We analyze the impact of temporal and spatial information on the performance of the resulting predictor.
- We train and validate the predictor by using 12 months of TT data collected by a BTMS deployed on the SE-30 and A-49 highways (Seville, Spain).

## 3. Materials and Methods

### 3.1. Previous Work

Our objective is to construct a short-term LOS predictor, exploiting the temporal and spatial components of traffic. In order to reach this objective, we take the basis of our previous work in [13], in which we built an LOS predictor by using a RUSBoost classifier fed with ATT data corresponding to the same instant of prediction. This classifier was capable of predicting LOS at 15 min in the future with an average 82.8% recall, which reached 92.5% predicting heavy congestions (LOS F). Despite these good results, the classifier reduced its performance when predicting intermediate LOS due to the instability that traffic shows under these circumstances. Consequently, we now focus on improving these capabilities by using temporal and spatial information. Let us present a brief overview of the first predictor we constructed; please visit [13] for further details.

TT is a statistical variable that shows the representative time that vehicles required to traverse a stretch at a given time. We can define two types of TT: ATT, which is the only measurement that a real-time system can provide, and DTT, which is the variable we seek for in order to inform the driver about the state of traffic that he expects to find on the stretch, when he is still at the origin. Given that TT is inherently affected by the specific features of the stretch under study, such as length or maximum speed limit, we moved to predicting LOS. LOS provides standardized information about the traffic state, ranging from free-flow (LOS A) to heavy congestion (LOS F), as it is defined in the Highway Capacity Manual [10].

As the base technology for our classifier we used an RUSBoost classifier [35], which is capable of dealing with unbalanced datasets as is the case of LOS, with evident majority (LOS A) and minority classes. RUSBoost works with partitions of the complete dataset including the number of samples in the minority class and iterates (60 times) the basic process to incorporate every sample in the overall training phase. Each step in this training phase employs a weak learn algorithm, a standard regression tree [36] in our case. The output of every step is a suboptimal model, which is then applied to the complete dataset in order to calculate the corresponding pseudo-loss and assign a new set of weights to every sample. The final result is a data-driven model for each link in the network and prediction horizon.

### 3.2. Empirical Data

**Figure 1:**
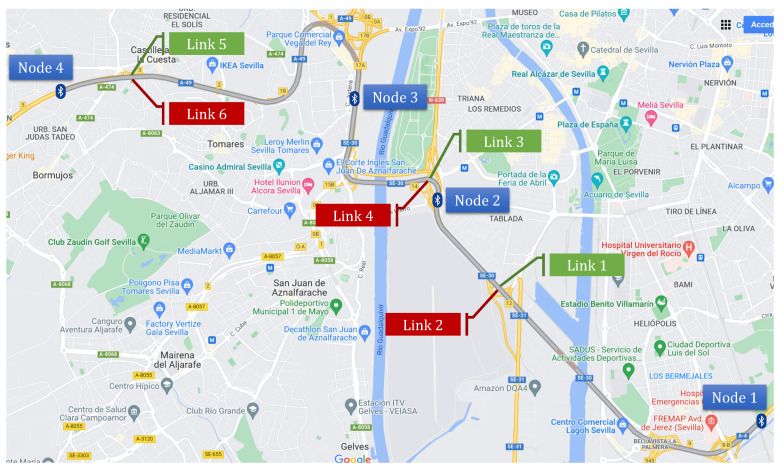
Nodes and links in the BTMS deployed in Seville (Spain).

The LOS predictor we aim to construct consumes ATT data. In our case, these data are collected by a BTMS deployed on the SE-30 and A-49 expressways in Seville (Spain), where we installed 4 Bluetooth identifiers, which define 6 links shown in Figure 1. We applied the TT calculation described in [9] over 100 million vehicle identifications produced by the BTMS during 2017 (12 months), which resulted in 525,600 1-min DTT and ATT values for every link.

### 3.3. Problem Statement

This work aims at constructing LOS predictors based on current and past ATT values, captured in the *L* links forming the road network. Let *t* be the moment of prediction and $t^{\prime }$ the prediction horizon; thus, at *t* we need to predict the specific LOS that will be observed by vehicles starting a particular link *k* at time $t+t^{\prime }$. In order to perform this prediction, the classifier consumes ATT values collected in every link *l* in the network (l=1,…,L) at times $p_{u}$ previous to *t* ($p_{u}<t$). Thus, for a given prediction time *t*, we construct *L* sets of input data

(1) $$ATT_{l}\left(t\right)=\left\{ATT_{l}\left(t\right),ATT_{l}\left(t-p_{1}\right),\ldots ,ATT_{l}\left(t-p_{n}\right)\right\},$$

where *n* is the number of *ATT* values previous to the time of prediction *t*. Consequently, the resulting feature space is $X\left(t\right)=ATT_{1}\left(t\right)\cup ATT_{2}\left(t\right)\cup \ldots \cup ATT_{L}\left(t\right)$.

The complete dataset *S* is then formed by samples

(2) $$({\vec{x}}_{k}\left(t\right),y_{i}\left(t+t^{\prime }\right)),$$

where ${\vec{x}}_{k}\left(t\right)\in X\left(t\right)$ and $y_{i}\left(t+t^{\prime }\right)\in Y=\left\{A,B,C,D,E,F\right\}$ is the LOS actually observed in link *k* at time $t+t^{\prime }$. In our case, *S* contains 525,600 samples per link, summing up to 3,153,600 in total.

### 3.4. Configuration of Predictors

We set up a maximum separation of 55 min between any temporal feature and the prediction horizon, which resulted in 27 predictors: 10 for 5-min prediction horizons ($t^{\prime }=5$ min), 9 for 10-min prediction horizons ($t^{\prime }=10$ min), and 8 for 15-min prediction horizons ($t^{\prime }=15$ min). As an illustrative example, the first predictor for $t^{\prime }=5$ min. was fed with ATT values registered on each link *l* at *t* and t−5, i.e., $\left\{ATT_{l}\left(t\right),ATT_{l}\left(t-5\right)\right\}$; and the tenth predictor for $t^{\prime }=5$ consumed input values $\left\{ATT_{l}\left(t\right),ATT_{l}\left(t-5\right),\ldots ,ATT_{l}\left(t-50\right)\right\}$.

As we showed in our previous work [13], traffic is deeply influenced by the type of day in the week, which resulted in a better performance of the predictor we constructed separating the sample data into four categories: Monday (Mon), Tuesday-Wednesday-Thursday (Tu/We/Th), Friday (Fri), and Saturday-Sunday-holiday (Sa/Su/ho). We built the new 27 predictors taking this same approach in order to compare their results to the best previous performance. In addition, we kept a fixed configuration for every predictor so that their results were not impacted by this factor. Consequently, every predictor used a standard decision tree [36] as the RUSBoost weak learn classifier, which was iterated 60 times, with a learning rate of 0.3. Finally, we selected 128 splits for categories Mon, Fri, and Sa/Su/ho and 196 splits for category Tu/We/Th, according to the feature space we managed. In order to validate the obtained results, we performed 5 independent runs of 5-fold cross validation. Thus, we split the dataset into 5 different partitions, using 4 of them to train the classifiers and the remaining one for testing. We analyzed the performance of these predictors by using recall as the relevant metric, given the imbalance shown within the set of classes [37] and its column-based nature from the perspective of the corresponding confusion matrices. We calculated recall values for each LOS and the result for every link, prediction horizon, and type of day.

## 4. Results

The 27 predictors were trained and validated by using ATT data captured by the BTMS deployed on the SE-30 highway in Seville, as we described in Section 3. Given the space limitations, for each link, prediction horizon, and type of day, we select the predictor with the best performance, which corresponds to a specific set of input features, i.e, how far in time from the time of prediction *t* we take ATT values. In order to complete this information, in Section 4.3 we will perform an analysis of the evolution of the prediction performance with the sequential addition of temporal features, in order to extract overall conclusions about the decision-making process in the design of LOS classifiers.

### 4.1. Performance Results

Table 1 shows the recall values we obtained for each LOS, using the best combination of past temporal information in all 6 links in the network. We can observe that the overall performance of the proposed predictor reaches 87.2% and rises above 70% for every LOS, even at 15-min prediction horizons. In addition, it is remarkable that the classifier shows a noticeable performance not only for free-flow (LOS A) and congestion (LOS F), which reach more than 90%, but also for intermediate traffic states LOS D (88.3%) and LOS E (94.2%). Moreover, the recall values obtained for LOS B and LOS C exceed 73%, thus confirming that the addition of temporal data provides further information about unstable traffic states that the classifier uses to increase its overall performance. Finally, let us mention that, as expected, performance degrades as we increase the prediction horizon; however, this degradation is soft, which supports the technical viability of the solution.

In order to provide the complete and detailed information about the performance of the predictors, Table 2 shows the recall values we obtained for each combination of link, prediction horizon, and type of day. In addition, it includes the total recall for the link and prediction horizon as the average performance reached on the set of types of day. Observing the particularized information, we can conclude that the overall performance is maintained throughout the links. This means that the average values in Table 1 are not biased by an outstanding performance in just a limited subset of links. The only exception that we notice is Link 3, in which LOS B and LOS C show lower recall values than the rest, even at a 5-min prediction horizon. Link 3 is particularly complex given that it includes several accesses to an industrial and commercial area, which implies a high number of heavy trucks that generate micro-congestions that increase the instability of the intermediate LOS in this section of the road. Regarding the type of day, we can observe no clear differences in the classifiers’ performance, which in consequence, fundamentally depends on the existence of some kind of intrinsic pattern that the predictor can extract from the data.

### 4.2. Performance Comparison with Previous Results

Comparing our current and previous results in [13], we can conclude that temporal information about past traffic behavior provides the classifier with a more solid knowledge base to predict future LOS. Table 3 and Table 4 show the comparative results.

Specifically, including temporal information as input data to the LOS predictors, we have raised its overall recall in 4.3%, to reach 87.2% total. This increase is particularly relevant as it comes from an already noticeable performance. In addition, the highest improvement occurs in the LOS D prediction (6.4% increase), one of the intermediate traffic states that showed a performance below the average in our previous results. Progress is even more significant if we observe the detailed results per type of day and LOS, which increase up to 23.3%, registered for LOS E at Link 6, on weekdays (Tu/We/Th), with a prediction horizon of 5 min. Nevertheless, inserting temporal information is not the ultimate solution for a perfect LOS prediction. Despite the fact that it achieves an overall performance increase, especially in some of the intermediate classes (LOS D and LOS E), it seems unable to significantly improve the recall values for LOS B and LOS C in Link 3, which are still below 65%. We will address this specific issue and propose future research to correct it in the discussion in Section 5.

### 4.3. Impact of Temporal Components on Performance

Once we have confirmed that temporal information improves the performance of LOS predictors, we will analyze the specific impact it produces. To this end, we provide figures displaying the evolution of the performance of each of the 27 predictors. As we described in Section 3.4, for each prediction horizon ($t^{\prime }=5,10,15$), we constructed a sequence of LOS classifiers adding ATT values from the six links in the network to the prior set of input variables.

**Figure 2:**
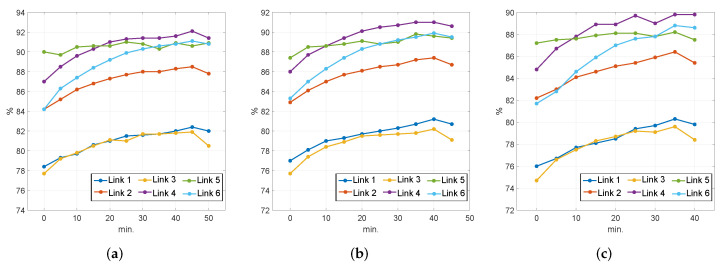
Evolution of the performance of predictors with $t^{\prime }=5$ min. (**a**) $t^{\prime }=10$ min. ATTs up to t−50 min. (**b**) and $t^{\prime }=15$ min. ATTs up to t−45 min. (**c**) depending on the input feature set. ATTs up to t−40 min.

Consequently, Figure 2a shows the evolution of the performance of classifiers for a 5-min prediction horizon for each of the six links in the road network (in different colors). The vertical axis displays global recall values while the horizontal axis refers to the times $p_{u}$ (u=0,1,…,10) previous to prediction time *t* in which we take ATT measurements. Thus, the leftmost points at $p_{0}=0$ act as the reference, showing the performance of the classifier with no temporal information, whose set of inputs is

(3) $$ATT^{0}\left(t\right)=\left\{ATT_{1}\left(t\right),ATT_{2}\left(t\right),\ldots ,ATT_{6}\left(t\right)\right\}.$$

The next group of points on each curve ($p_{1}=5$) represent the performance of the second classifier, which is fed with the input set

(4) $$ATT^{5}\left(t\right)=ATT^{0}\cup \left\{ATT_{1}\left(t-5\right),ATT_{2}\left(t-5\right),\ldots ,ATT_{6}\left(t-5\right)\right\}.$$

Subsequently, the last group of points on each curve show the recall achieved by the tenth classifier, which was built with the set of inputs

(5) $$ATT^{50}=ATT^{0}\cup ATT^{5}\cup \ldots \cup \left\{ATT_{1}\left(t-50\right),ATT_{2}\left(t-50\right),\ldots ATT_{6}\left(t-50\right)\right\}.$$

Accordingly, Figure 2b,c shows the evolution of the performance of classifiers for 10- and 15-min prediction horizons. In all three figures we can observe how the addition of new temporal components rapidly improves the performance up to a point where the curve becomes flat. Furthermore, it is noticeable that from a point in time onwards, extending the input feature set results in a decay in the performance of the LOS predictor. In fact, most of the time this decay occurs when the last ATT values we insert are 50 min away from the prediction horizon $t^{\prime }$: 45 min for $t^{\prime }=5$ (Figure 2a), 40 min for $t^{\prime }=10$ (Figure 2b), and 35 min for $t^{\prime }=15$ (Figure 2c). Note that this observation applies to the great majority of links, and the exceptions imply minimal differences in the recall values. Consequently, this suggests that the temporal components of ATT may lose predictive capabilities beyond a time separation of 50 min from the prediction horizon, which can be used as a sensible design principle for constructing LOS predictors based on ATT measurements.

## 5. Discussion

Our previous findings in [13] showed that spatial information not only from preceding and succeeding links, but also from those in the opposite direction, contributed to an increase in the LOS predictor’s performance. The work we have just presented extends those results and explores the predictive capabilities of traffic’s temporal components in order to improve the overall performance of LOS classifiers by selecting the appropriate set of input features. In this section, we discuss several relevant issues that derive from the results we have obtained and further strands of our work.

### 5.1. Predictor Importance

**Figure 3:**
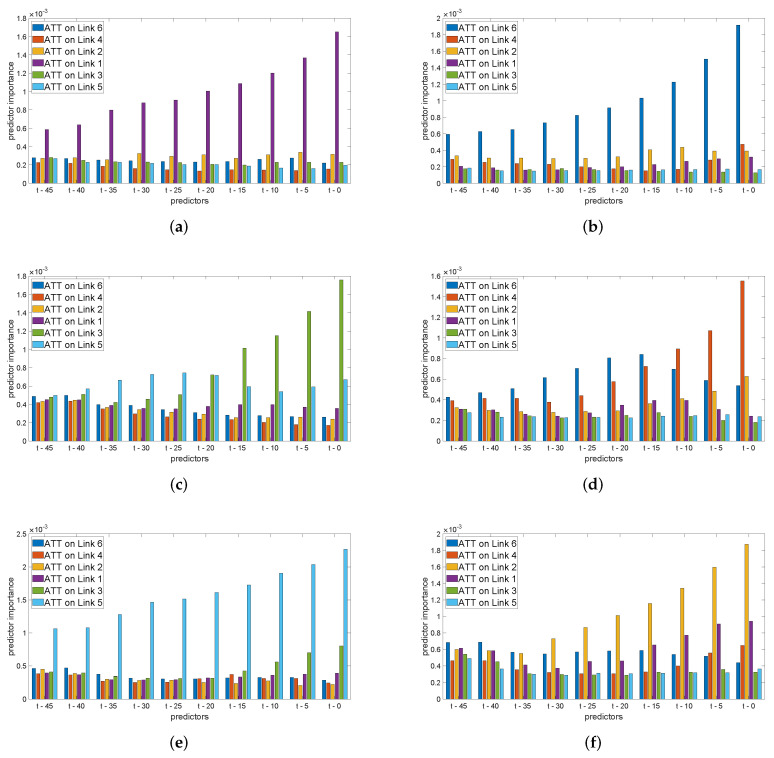
Predictor importance in classifiers with a 5-min prediction horizon on Mondays and ATT input features up to t−45 min. (**a**) Link 1. (**b**) Link 6. (**c**) Link 3. (**d**) Link 4. (**e**) Link 5. (**f**) Link 2.

We have performed a posteriori analyses regarding the predictor importance of every temporal component that the classifier used. This study provides us with information about which specific features within the overall set have played major roles and which others had smaller contributions to the overall result. To this end, in Figure 3 we display the predictor importance of each temporal feature that fed LOS classifiers applied to every link on Mondays, with a 5-min prediction horizon, and a set of previous ATT values captured up to 45 min from the time of prediction. The vertical bars in these figures are ordered in increasing time; furthermore within each group of features, links are organized by their direction on the road: southwards (links 6, 4, and 2) and northwards (links 1, 3, and 5) so as to facilitate the visualization of the impact of temporal components of the traffic on the same and opposite directions.

As we can observe in all six graphs in Figure 3, as expected, the most important feature is always the ATT on the link under study, at the time of prediction *t*. After it, the temporal information on the link itself keeps its relevance up until a point in time and the specific predictor built for each link shows distinct behaviors that do not present a direct relation with the physical configuration of the road network. Specifically, the starting links on the northbound (link 1) and southbound (link 6) sides of the expressway fundamentally take information from their own traffic as their most important features; however, the additional information the former includes comes from link 2 (which is in the opposite direction and has no direct connection to it), whereas the latter prefers information from links 2 and 4 (both in its direction). Regarding the central links 3 and 4, the most relevant information is taken from themselves and, respectively, from the succeeding (link 5) and preceding (link 6) stretches. On their part, the final northbound stretch (Link 5) primarily uses information from itself and the anterior (link 3), whereas the final southbound stretch (link 2) merges information from itself, link 1 (on the opposite direction), and link 6 (in its direction, but excluding its immediate precedent from the top 10 most important features).

These disjointed behaviors suggest that the learning process each predictor performs is capable of extracting correlations that are deeply hidden in the data, often presenting no straightforward connection to the real physical road network they work on. This is a clear advantage of data-driven approaches, but on the negative side, it requires further insight in order to come up with the exact operation of the classifier in order to reach a comprehensive knowledge about the intrinsic nature of traffic prediction.

**Figure 4:**
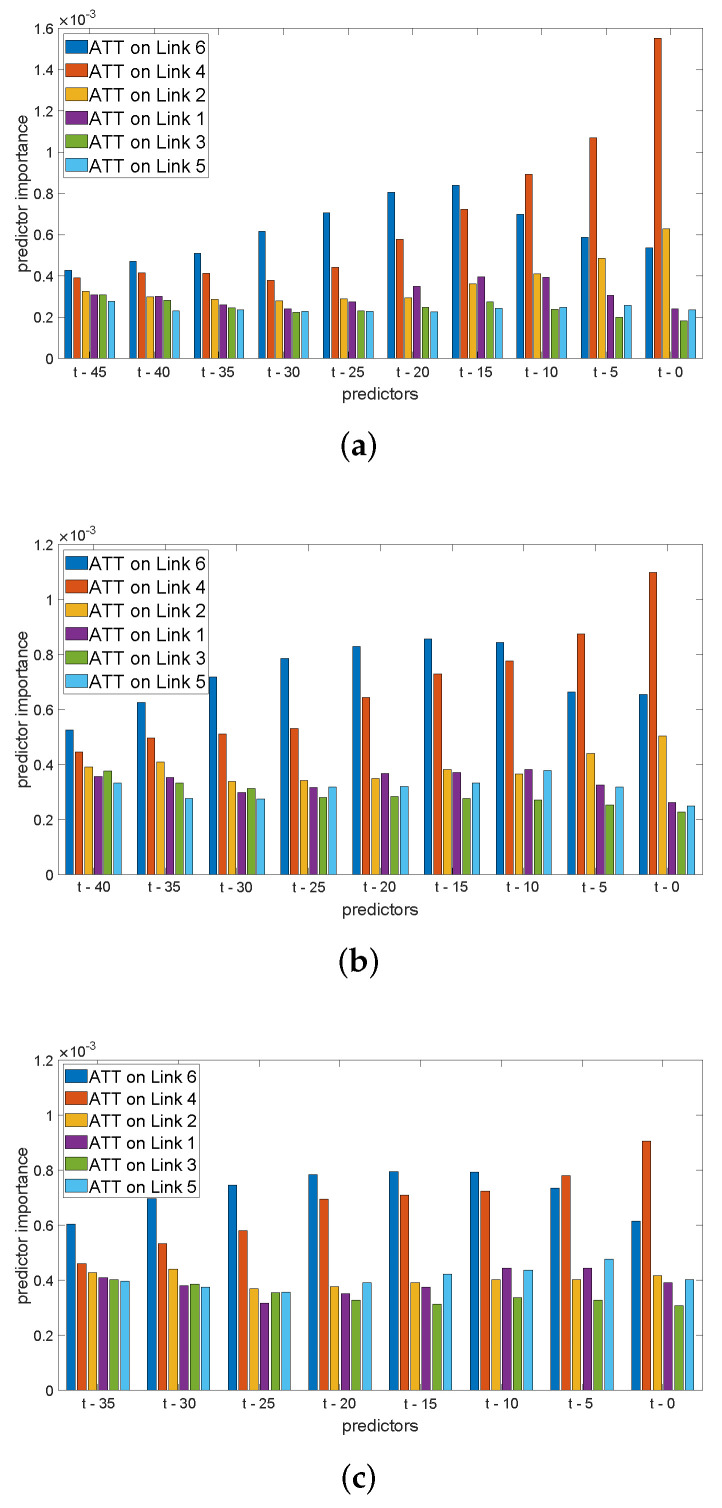
Predictor importance in classifiers at link 4, on Mondays. (**a**) ATTs up to t−45 min.; $t^{\prime }=5$ min. (**b**) ATTs up to t−40 min.; $t^{\prime }=10$ min. (**c**) ATTs up to t−35 min.; $t^{\prime }=15$ min.

In order to extend the prior study and present the evolution of each predictor importance with the prediction horizon $t^{\prime }$, in Figure 4 we particularize the predictor importance graphs to Link 4, with $t^{\prime }=5$, $t^{\prime }=10$, and $t^{\prime }=15$ and ATT input values up to 45, 40, and 35 min from the time of prediction respectively. In this case, we can observe that traffic information from the link under study loses importance as we increase the prediction horizon, and data from other links take a predominant role. In the light of these results, we can conclude that we require a combination of temporal and spatial components of traffic that do not always reflect an evident connection to the underlying physical structure of the road network.

### 5.2. Feature Selection

The predictor importance analysis led us to wonder whether we could extract that same knowledge about the predictive capabilities of temporal and spatial features prior to the training of the LOS classifiers. With this aim, we built and ran several feature selection algorithms in order to obtain those variables that could potentially improve the overall results and optimize the operational costs of the predictor. To this end, we tested two different filter methods, Relief [38] and MRMR [39], to find correlations with the desired output and redundancies among the set of input features. Nevertheless, we observed that first, the selected features depend on the classifier that we eventually chose, and second, their results did not match to those obtained from the predictor importance provided by the classifier itself. Thus, we abandoned this approach as an a priori way to select the potential optimum set of features.

### 5.3. Other Variables Produced by a BTMS

Finally, we also performed a battery of tests including other variables we could extract from a BTMS: vehicle count and residence time. The inherent nature of the BTMS makes it inappropriate for counting vehicles, given that it is always floored by the fact that it needs every vehicle to be equipped with a Bluetooth hands-free device in order to be detected. At present, most manufacturers incorporate this functionality, but there are still older models that do not. In any case, even if every vehicle had a Bluetooth device, there would always exist the probability of not detecting them as they traverse the zone of coverage. However, some level of counting is available from a BTMS whose trend could embed some useful information about the expected traffic behavior. We can address two types of BTMS vehicle counting: (i) associated to a node, considering any vehicle despite its direction; and (ii) associated to a stretch, selecting those vehicles that actually traversed the stretch under study registering a coherent TT.

On the other hand, given the zone-based nature of a BTMS, it is capable of detecting vehicles more than once as they traverse each node’s area of coverage, thus creating a multiple detection. We define residence time as the time elapsed between the first and last detections, whenever a multiple is produced. Thus we integrate all the individual residence times corresponding to vehicles within a certain slot in order to come up with a new traffic variable that somehow relates to the level of congestion on a stretch.

However, incorporating these two variables to the feature set did not improve the performance of the LOS predictors. This fact was confirmed by the subsequent predictor importance analysis, which highlighted the negligible contribution of vehicle counts and residence time to the internal construction of the classifier. The most apparent reasons for this observation are the low accuracy of the BTMS vehicle count and the insufficient number of multiple detections that eventually produced residence time measurements. In any case, other technologies such as inductive loops or radars, which are capable of providing far more accurate vehicle counts, may still be potentially useful for prediction purposes.

## 6. Conclusions

In this paper, we have presented an LOS predictor capable of exploiting the temporal and spatial components of traffic information, reaching an overall recall of 87.2%, with prediction horizons of up to 15 min. The predictors used 12 months of ATT data recorded by a BTMS in real operation on the SE-30 and A-49 highways in Seville, Spain. We have proven that temporal information increases the predictor’s global performance, particularly in those intermediate traffic states, which are especially complex due to their intrinsic instability. We have provided evidence about the evolution of the performance with the sequential addition of temporal components to the feature set, which first shows a rapid growth, followed by a reduction in the slope, and a final decay. This last decline occurs approximately when the input data is 50 min away from the prediction horizon, which constitutes a basic design principle. In addition, we have extended the study on the impact of temporal components on the construction of the LOS classifier by analyzing the predictor importance. This study indicated that there is no evident connection between the predominant input features and the underlying physical road network.

We are currently broadening this investigation, focusing on the internal process of the LOS predictor construction with a twofold objective: to further extend its current performance, and to achieve a deeper insight on the underlying mechanics of artificial intelligence applied to traffic.

## Figures and Tables

**Table 1:** Global performance results.

Horizon	LOS A	LOS B	LOS C	LOS D	LOS E	LOS F	Total
5 min	96.2%	77.0%	76.6%	88.8%	94.7%	97.0%	88.4%
10 min	95.0%	72.9%	75.3%	88.3%	94.3%	96.7%	87.1%
15 min	93.8%	70.2%	74.3%	87.9%	93.8%	96.3%	86.1%
**Total**	95.0%	73.4%	75.4%	88.3%	94.2%	96.6%	87.2%

**Table 2:** Detailed performance results.

**Horizon**	**Day**	**Link 1**						**Link 2**					
		**A**	**B**	**C**	**D**	**E**	**F**	**A**	**B**	**C**	**D**	**E**	**F**
5 min	**Total**	94.1%	74.4%	64.1%	77.2%	91.4%	94.3%	94.7%	80.5%	72.1%	90.5%	97.1%	98.3%
	Mon	93.8%	64.5%	59.7%	65.2%	94.4%	98.1%	93.5%	77.2%	66.1%	88.3%	98.9%	100%
	Tu/We/Th	91.6%	65.1%	45.9%	66.2%	90.6%	93.9%	92.5%	69.2%	56.7%	86.4%	96.0%	98.6%
	Fri	92.3%	75.8%	51.6%	77.4%	80.5%	90.9%	94.0%	76.7%	66.5%	87.2%	96.5%	96.2%
	Sa/Su/ho	98.7%	92.0%	99.1%	100%	100%	−	99.0%	98.8%	99.2%	100%	−	−
10 min	**Total**	92.9%	70.1%	64.4%	76.3%	90.9%	94.2%	93.4%	77.2%	69.8%	90.5%	96.5%	98.5%
	Mon	92.5%	59.5%	60.1%	64.7%	95.2%	97.8%	92.2%	74.4%	62.8%	88.6%	98.1%	100%
	Tu/We/Th	89.8%	59.2%	45.8%	63.8%	89.8%	93.2%	90.6%	63.4%	53.7%	85.7%	96.3%	98.1%
	Fri	91.6%	70.4%	53.1%	76.8%	78.7%	91.4%	92.2%	72.3%	63.6%	87.9%	95.0%	97.3%
	Sa/Su/ho	97.6%	91.4%	98.7%	100%	100%	−	98.6%	98.8%	99.1%	100%	−	−
15 min	**Total**	91.8%	68.0%	64.7%	76.0%	89.2%	94.0%	92.2%	75.9%	67.7%	89.4%	96.1%	98.3%
	Mon	91.4%	57.7%	58.2%	65.6%	93.3%	98.7%	90.8%	74.6%	60.3%	88.7%	98.1%	99.4%
	Tu/We/Th	88.3%	55.9%	45.0%	63.3%	87.9%	92.7%	89.1%	61.1%	49.0%	85.8%	95.7%	98.1%
	Fri	91.0%	67.5%	56.7%	75.3%	75.7%	90.6%	90.9%	69.2%	62.1%	83.4%	94.5%	97.5%
	Sa/Su/ho	96.5%	90.7%	98.7%	100%	100%	−	98.0%	98.8%	99.2%	100%	−	−
**Horizon**	**Day**	**Link 3**						**Link 4**					
		**A**	**B**	**C**	**D**	**E**	**F**	**A**	**B**	**C**	**D**	**E**	**F**
5 min	**Total**	95.8%	58.4%	62.6%	84.4%	96.1%	98.6%	99.0%	89.6%	89.2%	92.0%	90.6%	93.9%
	Mon	94.8%	61.1%	60.7%	88.1%	95.8%	96.5%	99.1%	94.5%	91.7%	91.4%	93.2%	92.2%
	Tu/We/Th	93.7%	47.3%	59.6%	82.4%	97.0%	97.7%	98.7%	78.5%	78.1%	80.8%	85.3%	91.4%
	Fri	96.0%	55.8%	54.4%	70.3%	92.9%	100%	98.3%	87.4%	86.9%	95.9%	93.3%	98.1%
	Sa/Su/ho	98.6%	69.4%	75.6%	96.6%	98.9%	100%	99.8%	98.2%	100%	100%	−	−
10 min	**Total**	94.0%	53.4%	60.0%	83.7%	96.0%	97.6%	98.6%	86.3%	88.6%	92.7%	90.3%	94.0%
	Mon	92.5%	54.6%	58.3%	88.3%	95.1%	96.0%	98.9%	93.0%	93.3%	91.6%	91.4%	96.1%
	Tu/We/Th	90.9%	40.8%	56.7%	82.6%	98.0%	96.0%	98.4%	71.0%	73.2%	85.0%	84.4%	88.7%
	Fri	94.6%	49.4%	51.8%	67.8%	93.0%	98.4%	97.3%	83.1%	88.0%	94.1%	95.0%	97.2%
	Sa/Su/ho	97.9%	68.7%	73.2%	96.3%	97.7%	100%	99.7%	98.1%	100%	100%	−	−
15 min	**Total**	92.5%	51.4%	58.5%	83.5%	96.8%	98.3%	98.1%	83.3%	87.1%	93.4%	88.9%	92.5%
	Mon	90.5%	53.2%	57.8%	88.6%	96.2%	97.0%	98.7%	93.9%	93.0%	92.9%	93.1%	94.6%
	Tu/We/Th	88.4%	38.3%	54.1%	81.9%	96.8%	98.3%	98.1%	62.2%	68.1%	86.2%	80.4%	85.5%
	Fri	93.7%	48.0%	49.8%	67.3%	94.1%	97.8%	95.9%	79.5%	87.3%	94.7%	93.3%	97.4%
	Sa/Su/ho	97.3%	65.9%	72.5%	96.3%	100%	100%	99.6%	97.6%	100%	100%	−	−
**Horizon**	**Day**	**Link 5**						**Link 6**					
		**A**	**B**	**C**	**D**	**E**	**F**	**A**	**B**	**C**	**D**	**E**	**F**
5 min	**Total**	96.3%	73.4%	84.9%	98.3%	99.8%	100%	97.0%	86.0%	86.9%	90.1%	93.0%	96.7%
	Mon	96.7%	79.1%	99.3%	99.3%	−	−	96.0%	89.4%	85.5%	85.8%	87.7%	92.6%
	Tu/We/Th	93.8%	64.9%	73.3%	96.2%	100%	100%	97.1%	76.2%	78.2%	81.3%	89.0%	96.5%
	Fri	96.1%	66.1%	81.2%	97.7%	99.5%	100%	96.4%	83.4%	85.1%	93.4%	96.2%	97.6%
	Sa/Su/ho	98.6%	83.4%	86.0%	100%	100%	100%	98.5%	95.0%	98.9%	100%	99.2%	100%
10 min	**Total**	95.1%	67.7%	82.7%	97.9%	99.6%	100%	95.9%	82.7%	96.4%	88.5%	92.2%	95.9%
	Mon	95.1%	76.9%	98.9%	99.3%	−	−	94.7%	87.7%	86.1%	83.2%	86.8%	90.2%
	Tu/We/Th	91.9%	58.4%	69.8%	95.7%	99.3%	100%	96.2%	69.5%	74.5%	76.7%	88.1%	96.3%
	Fri	95.0%	60.9%	75.9%	97.7%	99.5%	100%	94.7%	80.7%	86.2%	94.0%	94.8%	97.2%
	Sa/Su/ho	98.4%	74.8%	86.1%	98.9%	100%	100%	98.0%	92.9%	98.9%	100%	99.2%	100%
15 min	**Total**	93.5%	63.0%	82.2%	97.9%	99.6%	98.4%	94.8%	79.9%	85.6%	87.0%	92.1%	96.0%
	Mon	93.3%	73.4%	98.8%	99.7%	−	−	93.7%	84.0%	88.9%	81.9%	88.5%	91.8%
	Tu/We/Th	89.7%	51.9%	69.0%	95.0%	99.3%	100%	95.3%	63.4%	72.1%	74.1%	87.2%	95.2%
	Fri	93.6%	55.7%	74.3%	97.9%	99.5%	100%	92.9%	80.0%	83.4%	92.2%	93.6%	97.0%
	Sa/Su/ho	97.4%	70.9%	86.8%	98.9%	100%	95.2%	97.4%	92.1%	97.9%	100%	99.2%	100%

**Table 3:** Improvements with respect to prior results in each LOS.

Horizon	LOS A		LOS B		LOS C	
	Previous	Current	Previous	Current	Previous	Current
5 min	96.0%	96.2%	72.5%	77.0%	71.4%	76.6%
10 min	94.8%	95.0%	68.1%	72.9%	69.3%	75.3%
15 min	93.5%	93.8%	65.4%	70.2%	68.9%	74.3%
**Total**	94.8%	95.0%	68.7%	73.4%	69.9%	75.4%
**Horizon**	**LOS D**		**LOS E**		**LOS F**	
	Previous	Current	Previous	Current	Previous	Current
5 min	82.7%	88.8%	89.7%	94.7%	92.5%	97.0%
10 min	81.9%	88.3%	89.1%	94.3%	93.0%	96.7%
15 min	82.0%	87.9%	88.4%	93.8%	92.0%	96.3%
**Total**	82.2%	88.3%	89.0%	94.2%	92.5%	96.6%

**Table 4:** Global Improvements with respect to prior results.

**Horizon**	**Total**	
	Previous	Current
5 min	84.1%	88.4%
10 min	82.7%	87.1%
15 min	81.7%	86.1%
**Total**	82.8%	87.2%

## Data Availability

Not applicable.
